# The Effect of Probiotics on Preterm Birth Rates in Pregnant Women After a Threatened Preterm Birth Episode (The PROPEV Trial)

**DOI:** 10.3390/biomedicines13051141

**Published:** 2025-05-08

**Authors:** Ester del Barco, Leidy-Alejandra G. Molano, Mireia Vargas, Marta Miserachs, Linda Puerto, Carmen Garrido-Giménez, Zaida Soler, Begoña Muñoz, Laia Pratcorona, Sonia Rimbaut, Mercè Vidal, Marta Dalmau, Alba Casellas, Elena Carreras, Chaysavanh Manichanh, Maria Goya

**Affiliations:** 1 Maternal-Fetal Medicine Unit, Department of Obstetrics, Vall d’Hebron Barcelona Hospital Campus, Universitat Autònoma de Barcelona, 08035 Barcelona, Spainmaria.goya@vallhebron.cat (M.G.); 2 Microbiome Lab, Vall d’Hebron Institut de Recerca (VHIR), Vall d’Hebron Barcelona Hospital Campus, Passeig Vall d’Hebron, 08035 Barcelona, Spain; 3 Obstetrics and Gynecology Department, Hospital Althaia, 08243 Manresa, Spain; 4 Obstetrics and Gynecology Department, Hospital Mútua de Terrassa, 08221 Terrassa, Spain; 5 High-Risk Obstetrics Unit, Obstetrics Department, Hospital Universitari de Tarragona Joan XXIII, 43005 Tarragona, Spain; 6 Maternal-Fetal Medicine Unit, Department of Obstetrics, Hospital de la Santa Creu i Sant Pau, 08025 Barcelona, Spain; 7 Women and Perinatal Health Research Group, IR SANT PAU, RICORS-SAMID Network (RD21/0012), Instituto de Salud Carlos III, 28040 Madrid, Spain; 8 Obstetrics and Gynecology Department, Hospital Sant Joan de Reus, 43204 Reus, Spain; 9 Obstetrics and Gynecology Department, Hospital Germans Tries I Pujol, 08916 Badalona, Spain; 10 Obstetrics and Gynecology Department, Institut Dexeus, 08028 Barcelona, Spain; 11 Obstetrics and Gynecology Department, Hospital Sant Joan de Déu, 08950 Esplugues de Llobregat, Spain

**Keywords:** prematurity, preterm birth, microbiota, probiotics, *Lactobacillus*

## Abstract

**Introduction**: Preterm birth is the leading cause of perinatal mortality worldwide, with prevalence rates showing little reduction. Although mortality rates have decreased, morbidity rates remain concerningly high. In recent years, there has been a surge in studies examining the etiology, risk factors, and management of preterm birth. The use of vaginal probiotics in pregnant women at risk of preterm birth has garnered attention as a potential approach for improving perinatal outcomes and modulating the vaginal microbiota. However, the efficacy of this intervention remains unclear. Therefore, this study explored the impact of vaginal probiotics on perinatal outcomes and vaginal microbiota composition in pregnant women at risk of preterm birth. **Materials and Methods**: This was a randomized, prospective, longitudinal, double-blind, placebo-controlled, multicentric trial conducted across seven maternities in Spain from October 2017 to August 2022 in pregnant women at risk of preterm birth. Participants were randomly assigned to receive vaginal probiotics containing four lactobacilli strains or a placebo. The primary outcome was to explore a potential correlation between probiotic use among pregnant women at risk of preterm birth and the actual rate of preterm birth before 37 gestational weeks. Secondary outcomes included an evaluation of preterm birth rates, neonatal morbidity, the vaginal microbiota, and changes in the vaginal microbiota after receiving probiotics. Other secondary outcomes were identifying vaginal microbiota patterns associated with preterm birth and exploring potential therapeutic mechanisms involving probiotics. Trial registration: Clinicaltrials.gov, identifier: NCT03689166. **Results**: A total of 200 participants were included. Of those, birth data were obtained for 181 women. Demographics were similar between both groups. An analysis of perinatal outcomes found no significant differences in preterm birth rates, prematurity rates, gestational weeks at delivery, neonatal complications, time to birth, or latency time to delivery. Microbiota analysis showed no significant differences in vaginal microbiota changes between groups. No serious or unexpected adverse reactions were reported. **Conclusions**: There were no statistically significant differences for spontaneous preterm birth between pregnant women receiving probiotics and pregnant women receiving the placebo.

## 1. Introduction

Preterm birth (PTB) is the leading cause of neonatal death (within the first 28 days of life) and accounts for 27% of neonatal deaths worldwide [1,2]. The risk of neonatal death decreases as gestational age increases, but according to a non-linear relationship. In addition, PTB has severe consequences for preterm neonates, including increased morbidity and a greater rate of long-term consequences, such as impaired psychomotor development and increased risk of chronic diseases in adulthood [3]. The global rate of PTB is estimated to be approximately 11% (fluctuating by 5% in some European countries and 18% in some African countries) [4,5].

Approximately 70% of preterm births are spontaneous, either due to a risk of preterm birth (45%) or due to the premature rupture of membranes (25%); the remaining cases (30%) are a result of maternal or fetal conditions, such as multiple gestations, preeclampsia, antepartum hemorrhage, and fetal growth restriction [6].

Progress in recent years has allowed for an increase in the survival rate of neonates with very low birth weights. However, prematurity rates have not changed significantly [7], and increased survival is associated with many respiratory, visual, auditory, neurological, and cognitive complications, as well as certain psychosocial behaviors [8].

For this reason, recent trends have involved identifying PTB risk factors and developing prevention strategies.

Several studies have revealed the importance of vaginal microbiota composition in determining a healthy reproductive tract [9,10,11]. The vaginal microbiota is often categorized into five distinct community state types (CSTs), which reflect the predominance of a particular bacterium within the vagina. CSTs I, II, III, and V are defined by the dominance of *Lactobacillus crispatus*, *L. gasseri*, *L. iners*, and *L. jensenii,* respectively. By contrast, CST IV presents a highly diverse microbial composition, where no single bacterium is dominant. This community usually comprises various anaerobic species belonging to the genera of *Gardnerella*, *Prevotella*, *Fannyhessea*, *Mobiluncus*, *Annaerococcus*, and *Sneathia*. CSTs with an overall dominance of *Lactobacillus* species have been typically associated with healthy states. In contrast, non-*Lactobacillus* microbiomes are often linked to high levels of pro-inflammatory cytokines and adverse health outcomes, including spontaneous preterm birth (sPTB) [12,13,14,15]. However, CST III characterized by *L. iners* dominance has also been associated with a higher risk of sPTB, whereas *L. crispatus* dominance was found to have a protective effect [16,17,18].

One potentially new way to protect against PTB-mediated infection is using probiotic bacteria, particularly *Lactobacillus*. Probiotics, defined as living microorganisms that, in suitable amounts, benefit the health of the host, are being studied for their ability to replenish vaginal *Lactobacillus* and modulate immunity.

Several studies have determined the effectiveness of *Lactobacillus* spp. when dealing with vaginal pathogens such as *Gardnerella* vaginalis and other microorganisms involved in bacterial vaginosis [19,20,21,22].

Many strains of *Lactobacillus* have been evaluated, including *L. gasseri*, *L. acidophilus*, *L. reuteri*, *L. rhamnosus*, *L. paracasei*, *L. plantarum*, and so on [19,20,21,22,23,24,25,26,27], either separately or combined. Several strains of *Lactobacillus* given to women with bacterial vaginosis can reduce symptoms effectively, even when given as a single treatment [28], as well as decrease recurrence with adjuvant antibiotic treatment [29,30,31]. In addition, using probiotics during pregnancy and breastfeeding has been described by several authors as being a safe and effective method for improving the potential immunoprotective role of breast milk and preventing atopic eczema in the neonate [32,33].

Despite the growing interest in the role of the vaginal microbiota in pregnancy outcomes, there is a lack of randomized clinical trials specifically assessing the impact of vaginal probiotic administration in women with a threatened preterm labor episode. Most existing studies have focused on asymptomatic populations or earlier stages of gestation. Therefore, this study aims to address this gap by evaluating whether vaginal probiotics can modify the vaginal microbiota and reduce the incidence of spontaneous preterm birth in a high-risk, symptomatic population.

We hypothesized that vaginal administration of a four-strain probiotic in women with threatened preterm labor would reduce the risk of spontaneous preterm birth through the modulation of the vaginal microbiota.

The aim of this study was to determine whether the use of probiotics in patients at risk of spontaneous PTB reduces the rates of PTB before 37 weeks of gestation due to changes in the vaginal microbiota. This will also help us understand the pathophysiology of spontaneous PTB and the mechanisms regulating the vaginal microbiota in pregnant women.

The secondary study hypothesis was that pregnant women at risk of PTB will exhibit a distinct vaginal microbiota compared to pregnant women without a risk of PTB. Additionally, this study hypothesized that using probiotics will alter the vaginal microbiota of pregnant women at risk of PTB. Furthermore, it was expected that the incidence of PTB before 37 weeks of gestation in pregnant women at risk of PTB may decrease by at least 30% following the probiotic intervention.

This study focuses on the use of probiotics as a preventive strategy during the current pregnancy in women at high risk of preterm birth, with the aim of reducing adverse perinatal outcomes associated with this pregnancy, rather than as a long-term intervention strategy for future gestations.

## 2. Material and Methods

### 2.1. Study Design and Participants

This randomized, prospective, longitudinal, double-blind, placebo-controlled, multi-center trial was conducted at seven maternity hospitals across Spain between October 2017 and August 2022 (Hospital Universitari Vall d’Hebron, Hospital Universitari Sant Joan de Reus, Hospital de la Santa Creu i Sant Pau, Hospital Quirón Salud de Barcelona, Hospital Universitari Dexeus, Hospital Universitari Germans Trias i Pujol, and Hospital Universitari Joan XXIII de Tarragona).

Inclusion criteria at randomization were as follows: maternal age of 18 years or older; singleton gestation with a threatened preterm labor episode, defined as ≥4 regular uterine contractions in 30 min, which were confirmed both clinically and via cardiotocography, and associated with a cervical length ≤ 25 mm between 24 and 29 weeks of gestation and ≤15 mm between 30 and 34 weeks of gestation, as well as cervical dilation ≤ 2 cm; and a sonographically normal fetal morphology.

Exclusion criteria were as follow: multiple gestations, clinical chorioamnionitis (diagnosed according to Gibbs’ criteria), and cervical dilation > 2 cm.

### 2.2. Sample Size

For a significance level of 5%, the number of patients to be included to demonstrate a 30% reduction in the rate of PL < 37 weeks (50% of cases) is 103 cases in every group, with 80% power. This reduction was selected based on the anticipated proportion and the sample size appropriate for our hospital. A total of 103 patients were included in each follow-up group; patients were randomly assigned according to the inclusion and exclusion criteria outlined in the previous section, with a 1:1 ratio, forming two groups: one receiving a placebo (control group) and the other receiving probiotics (treatment group).

### 2.3. Procedures, Randomization, and Masking

Standard management of women at risk of PTB included hospitalization due to tocolytic treatment and fetal lung maturation. Vaginal, endocervical, urinary, and vagino-rectal SGB cultures, as well as occasional amniocentesis (according to each hospital’s protocol), were performed to exclude subclinical chorioamnionitis.

After considering the inclusion and exclusion criteria, a total of 103 patients were randomized according to a 1:1 ratio into two groups: an intervention group (group A) receiving probiotics and a control group (group B) receiving the placebo.

Randomization was performed using a random number list (RNL) generated by the Statistics Unit of Vall d’Hebron Research Institute (VHIR) and stored in an electronic notebook. The clinicians overseeing this study were blinded to the list details.

The placebo group functioned as the negative control, given that there is currently no established or approved probiotic treatment for the prevention of preterm birth. Therefore, a positive control group was not applicable in this study design.

The probiotic intervention consisted of a combination of 4 *Lactobacillus* strains (*Lactobacillus crispatus LBV88*, *Lactobacillus rhamnosus LBV96*, *Lactobacillus jensenii LBV116*, and *Lactobacillus gasseri LBV150*) with fructooligosaccharides in a capsule. The placebo consisted of maltodextrin (244 mg), a vegetable capsule (75 mg), and optionally magnesium stearate and silicon dioxide (6 mg) for a total capsule weight of 325 mg. Both the placebo and the probiotic capsules had an identical appearance. Treatment was administered vaginally.

Once the group had been assigned, the patients started the intervention with two vaginal capsules of the probiotic or placebo until 36.6 weeks of gestation. The maximum treatment duration was from 24.0 to 36.6 weeks of gestation, and the minimum treatment duration was from 34.6 to 36.6 weeks of gestation. Upon admission, treatment with vaginal progesterone was initiated, or a pessary was placed according to each hospital’s protocol.

Vaginal microbiota samples were collected from 60 patients from a subset of participants recruited at Vall d’Hebron Hospital, as part of a pre-specified microbiome sub-study. All participants were randomized based on the strict inclusion and exclusion criteria described above. The selection of this subgroup was based on logistical feasibility and laboratory availability for microbiome analysis at this center.

Both groups underwent the same follow-up protocol at each site: monthly visits until delivery or until 37 weeks of gestation. An abdominal ultrasound to assess fetal biometrics and a transvaginal ultrasound to assess cervical length were performed at each visit. Additionally, at Hospital Vall d’Hebron, a new analysis of the vaginal microbiota was performed one month after the start of the intervention.

Given that both groups underwent the same follow-up procedures and received capsules identical in appearance and administration schedule, the comparison between the groups under equivalent conditions provides a valid assessment of the effect (or lack thereof) of the probiotic intervention.

### 2.4. Outcomes

The primary outcome was to explore a potential correlation between the use of probiotics in pregnant women at risk of PTB and the actual PTB rate before 37 weeks of gestation. The secondary outcomes were to assess the rate of PTB < 28, 30, 32, and 34 weeks of gestation in both groups; to assess neonatal morbidity; to analyze the vaginal microbiota of pregnant women at risk of PTB; to assess changes in the vaginal microbiota after using probiotics; to identify vaginal microbiota patterns related to PTB; and to investigate a potential therapeutic mechanism involving probiotics for reducing the risk of PTB.

### 2.5. Statistical Analysis

Data were analyzed using the statistical package IBM SPSS Statistics, version 27.0 for Windows 19.0.

The efficacy analysis was conducted using an intention-to-treat approach. Patients in the study group were randomly assigned to one of the two intervention groups.

Secondly, re-sampling using the bootstrap method in which preterm births were randomly assigned in the same proportion as in the whole study population was performed. Average differences between the groups were calculated.

A descriptive analysis was conducted for the entire study population and for those who reached birth. For the entire study population, given that it was a randomized clinical trial and following the CONSORT guidelines [34], a hypothesis test was not conducted on baseline data because, by definition, both groups were equivalent except for the intervention. Therefore, any observed differences in descriptive measurements in the general criteria were attributed to the randomization procedure.

When comparing groups in terms of reaching birth and gestational age at birth, statistical hypotheses were assessed using the Chi-squared test or the Fisher test for qualitative variables and the Mann–Whitney U test for quantitative variables.

Birth and post-partum data were also collected. The results of all analyses were evaluated considering a type I error level set at 0.05. *p*-values are reported with four decimal places.

In the descriptive analysis, qualitative variables are expressed as the absolute number of cases and percentage in each category. Quantitative variables are expressed as the mean (standard deviation), the median (interquartile range), and the minimum and maximum.

**Ethics Statement**: The protocol was approved by the local Ethics Committee of each participating hospital (registration number: PR(AMI)359/2014 and date of issue: 1 December 2014). The study protocol was registered in Clinicaltrials.gov on 11 February 2018 (Identifier: NCT03689166 and URL address: https://clinicaltrials.gov/study/NCT03689166 (accessed on 16 September 2022)) [35]. All participants provided their written informed consent.

### 2.6. DNA Extraction and 16S rRNA Sequencing

Cervicovaginal swabs were collected in Deltalab tubes during a speculum examination (Deltalab Amies; Copan Italia, LQ Amines) and stored at −80 °C until they were processed. Before DNA extraction, the liquid content of the swab was transferred into a 2 mL tube with a screw tap and centrifuged at the maximum speed (18.213× *g*) for 15 min. After centrifugation, the supernatant was removed using a 200 μL pipette, and the remaining pellet in combination with the original swab was kept for further processing following the recommendations of the International Human Microbiome Standards [36] and as previously described [37,38]. The integrity and quality of the DNA were assessed using a NanoDrop ND-1000 spectrophotometer (Nucliber, Madrid, Spain) and a 1% agarose gel.

For profiling the microbial composition, genomic DNA was used to amplify the V4 region of the 16SrRNA gene using polymerase chain reaction (PCR). Sequences of the primers used for targeting the region were as follows: 5′-{AATGATACGGCGACCACCGAGATCTACACTATGGTAATTGT}{GTGCCAGCMGCCGCGGTAA}-3′ and 5′-{CAAGCA GAAGACGGCATACGAGAT} {Golay barcode}{AGTCAGTCAGCC} {GGACTACHVGGGTWTCTAAT}-3′ [38,39]. Amplicons were then purified using the QIAquick PCR Purification Kit (Qiagen, Barcelona, Spain) and pooled in equal concentrations. Pooled amplicons were purified a second time using HighPrepTM PCR magnetic beads (Magbio, Genomics Inc., Rockville, MD, USA) and sent to the Illumina MiSeq platform (UAB, Spain) for sequencing following the standard Illumina protocols [15]. Additionally, for microbial load determination, the V4 region of the 16S rRNA gene was amplified using real-time quantitative PCR (qPCR) following a previously described method [40].

### 2.7. Microbiota Analysis

At Vall d’Hebron Hospital, vaginal samples were collected to analyze the microbial composition from a selected group of 55 patients at randomization. Samples were collected from both groups at baseline and four weeks after starting the intervention.

The 16S rRNA raw sequence data were preprocessed using the QIIME2 (v.2020.2.0) bioinformatics pipeline [41]. The sequence reads were denoised, filtered out from chimeras, and de-replicated into amplicon sequence variants (ASVs) using the DADA2 tool, version 2020.2.0 [42]. Each ASV was taxonomically assigned to the species level using the QIIME2 naive-bayes feature classifier with GSR-DB 16S as the reference database [43]. The range of the n-gram parameter was set to [7], and the confidence threshold was set to “disable”.

Community state types (CSTs) were assigned to each sample based on the taxon with the largest abundance as previously described.

The multivariable association with linear models (MaAslin2, v.1.12.0) [44], ANCOM-BC2 (v. 2.0.2) [45], and the Wilcoxon signed-rank test were used to assess the differences in abundance between microbiota data and clinical variables. The use of different methodologies is highly recommended to increase the robustness of the results [46]. The following parameters were used to set up MaAslin2: normalization = 9“TSS”, transform = “LOG”, analysis_method = “LM”, standardize = “TRUE”, max_significance = 0.05, min_abundance = 0.0001, and min_prevalence = 0.1. ANCOM-BC2 was used with the following parameters: p_adj_method = “holm”, prv_ct = 0, struct_zero = “TRUE”, neg_lb = “TRUE”, alpha = 0.05, dunnet = “TRUE”, and trend = “TRUE”. Unlike MaAsLin2, in ANCOM-BC2, we did not include any filtering (prv_ct = 0) in order to identify potential structural zeros. Intervention with probiotics, the time point, and sPTB were included as fixed effects. Subject identification was added as a random effect.

Differences in microbial load and alpha diversity among groups were assessed using the non-parametric Wilcoxon signed-rank test (paired comparison). Alpha diversity indexes (Chao1, Shannon, and Simpson) were calculated using the vegan package (v. 2.6-4) in R [47]. The PERMANOVA test was used to analyze the associations between the microbial composition (beta diversity) and host factors using the adonis2 function of the vegan package with the following settings: setting permutation = 10,000, by = “terms”, and seed = 123. Beta diversities were calculated using Bray–Curtis dissimilarities between samples (calculated with the vegan package) and visualized using principal component analysis (PCA) ordinations.

Bacterial species were not removed from the analysis through cleaning processes. Additionally, no environmental controls or mock communities were used as controls for the microbiota analyses.

## 3. Results

**Chart 1:**
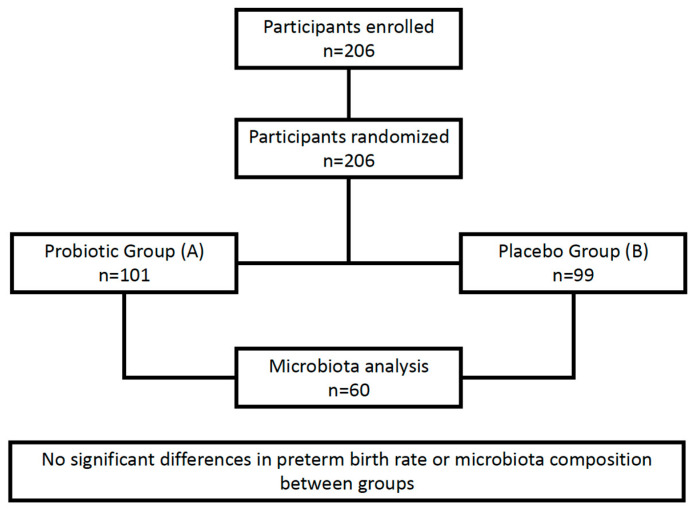
Flowchart of the PROPEV trial, illustrating the study design and participant progression through the trial phases.

During the recruitment period, 271 patients were assessed for eligibility, of whom 65 were excluded, mainly due to cervical dilation > 2 cm, multiple gestation, or clinical signs of chorioamnionitis. A total of 200 participants were recruited: 101 women (50.50%) were assigned to the intervention group (group A), and 99 (49.50%) were assigned to the placebo group (group B). Delivery data were available for 181 participants: 91 (50.28%) in group A and 90 (49.72%) in group B. Nineteen participants were lost during the follow-up period. The main reasons were loss of contact or delivery at a different hospital (Chart 1).

The demographic characteristics of all patients are shown in Table 1.

Although no statistically significant differences were observed for the primary outcome of preterm birth < 37 weeks, a trend toward a lower rate of preterm birth < 34 weeks was noted in the placebo group (17.8% vs. 9.0%, *p* = 0.0844), and a statistically significant difference was observed for preterm birth < 32 weeks (12.2% vs. 3.3%, *p* = 0.0260). However, these results should be interpreted with caution, as multiple comparisons were performed across various gestational age thresholds. After applying Bonferroni correction, the *p*-values did not retain statistical significance. Therefore, these findings may reflect chance variation rather than a true effect of the intervention and should be considered exploratory. Further studies with larger sample sizes are needed to confirm whether these trends are clinically meaningful (Table 2).

The composite outcome was defined as spontaneous premature births before 37 weeks of gestation, cases with a low birth weight, or the presence of any other neonatal complication (neonatal death, admission to NICU, hyaline membrane, cerebral hemorrhage, prenatal sepsis, or necrotizing enterocolitis). The distribution of neonatal morbidities was similar across both groups, and no statistically significant differences were observed between both groups (Table 3).

**Figure 1:**
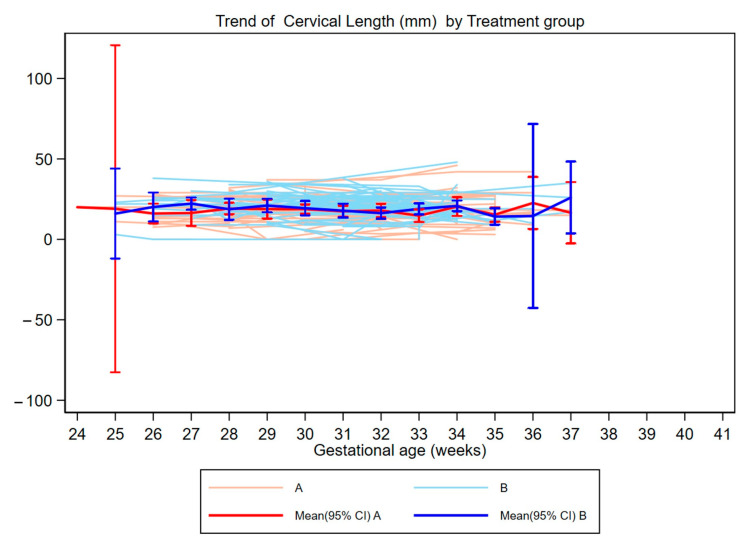
Individual trajectories of cervical length percentiles throughout pregnancy, grouped by intervention. Each line represents a single participant. Group A: probiotic treatment; Group B: placebo.

Furthermore, when we compared the evolution of cervical length throughout gestation in both groups, no differences were observed (Figure 1).

**Figure 2:**
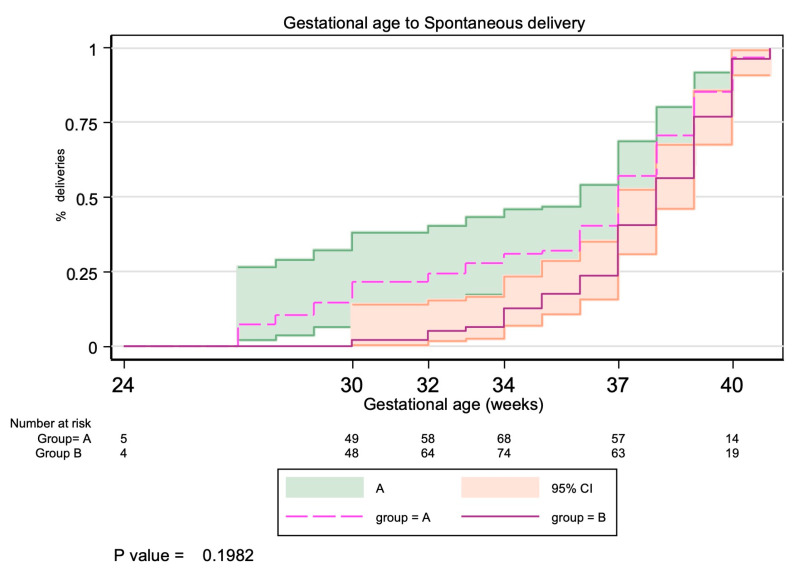
Kaplan–Meier analysis of time to spontaneous delivery (weeks) by intervention group. Group A: probiotic treatment; Group B: placebo. The x-axis displays selected gestational age milestones that are clinically relevant in obstetrics: 24 weeks (viability threshold), 30 and 32 weeks (key time points at which the risk of severe neonatal morbidity begins to decrease), 34 weeks (late preterm), 37 weeks (term), and 40 weeks (full-term pregnancy).

**Figure 3:**
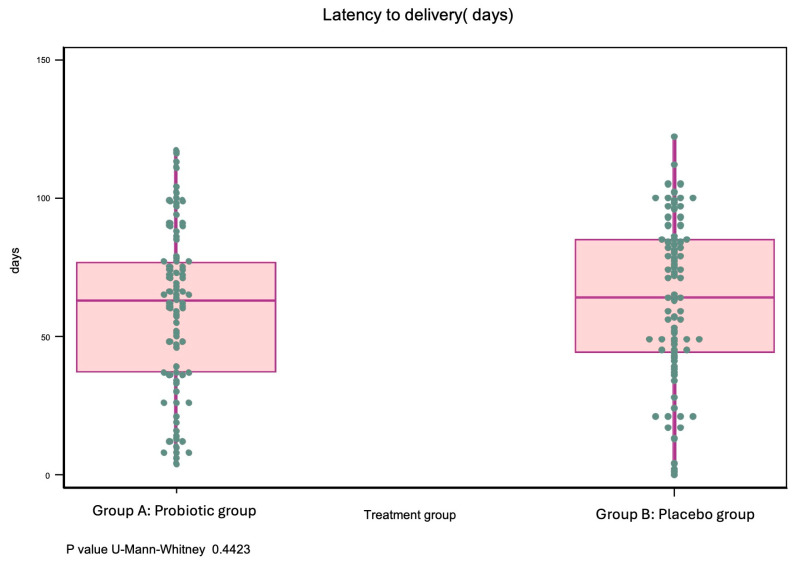
Latency time to delivery (in days) from randomization by intervention group. Group A: the probiotic group; Group B: the placebo group.

We performed a survival analysis to assess time to birth from 24 weeks of gestation, accounting for the fact that each woman entered the study at a different gestational age. Results are shown in Figure 2. Births induced before 37 weeks of gestation were removed from this analysis. A survival model for truncated data was used, comparing only women who remained at risk of preterm birth at each time point. This approach corrected for variability in the timing of study entry and follow-up across participants. No significant differences were observed between the probiotic and placebo groups (Figure 3).

### 3.1. Microbiota Analysis

One of the secondary endpoints was the modification of vaginal microbiota in pregnant women at risk of PTB receiving probiotics. To determine this endpoint, a sample of the vaginal microbiota was collected in patients recruited at Hospital Vall d’Hebron.

At the time of enrollment, the vaginal microbiota of high-risk pregnant women was predominantly composed of 10 genera, accounting for 98% of the total sequencing data. Among those genera, *Lactobacillus* was the most abundant (78.42%), followed by *Gardnerella* (9.2%), *Bifidobacterium* (4.3%), *Fannyhessea* (1.8%), and *Prevotella* (1.8%). The most common community state type (CST) was dominated by *L. iners* (45%, CST III), followed by *L. crispatus* (25%, CST I) and a highly diverse community (18%, CST IV). CST IV was mainly composed of *G. vaginalis* (38%), *B. dentium-moukalabense* (10.6%), *F. vaginae* (8.8%), and *B. longum* (8.8%). CST II, which was dominated by *L. gasseri*, was found in 25% of the participants, and only one participant was clustered into the CST V group, which was dominated by *L. jensenii*. No differences in the proportion of CSTs between groups was observed (*p*-value = 0.69, Fisher’s exact) before intervention (Table 4).

**Figure 4:**
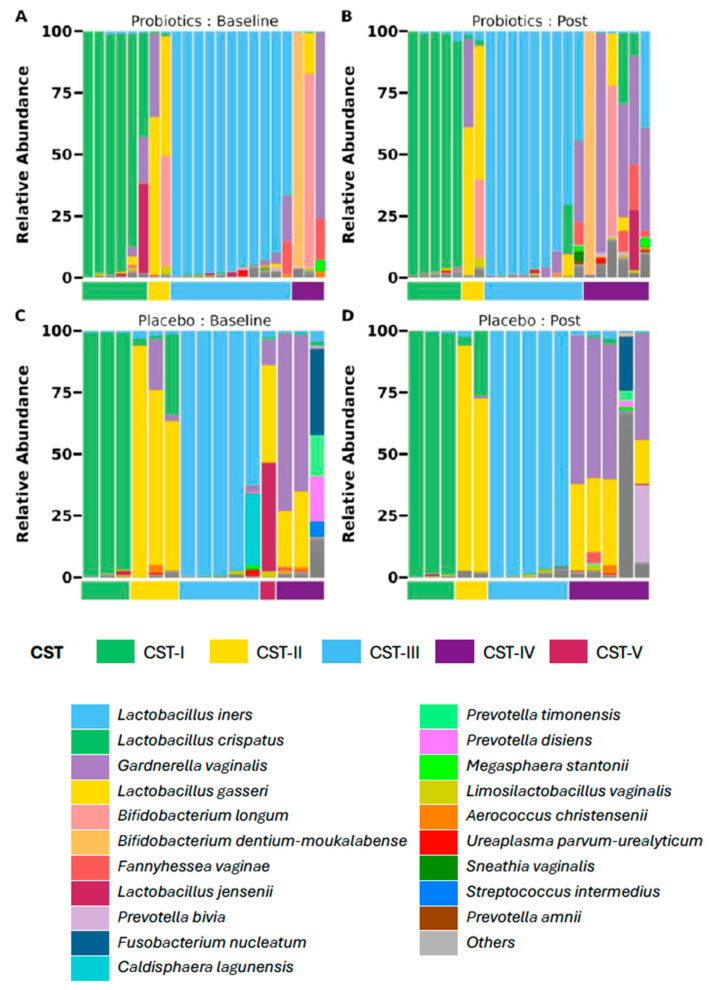
Vaginal microbial profiles before and after the intervention by study group. Each panel displays community state types (CSTs) and dominant bacterial species per participant at baseline (**A**,**C**) and after 4 weeks (**B**,**D**). Only paired samples are shown (probiotics: *n* = 22 and placebo: *n* = 15). **Top panels** (**A**,**B**): probiotic group (*n* = 22); **Bottom panels** (**C**,**D**) ): placebo group (*n* = 15).

After the intervention, the microbial profiles in both the placebo and probiotic groups remained stable (Figure 4). No significant differences were found in beta diversity, alpha diversity, or microbial load. Consistently, no differentially abundant genera or species were observed between women who received probiotics as compared to those who received the placebo, according to the MaAsLin2, ANCOM-BC2, and Wilcoxon tests. Interestingly, over the 4-week follow-up, although the CST III microbial profile remained predominant, CST IV (29%) was established as the second most predominant, shifting CST II (20%) to the third position (Table 4). This change is explained because six women had a CST shift, five of them shifting from *Lactobacillus*-dominated CST to CST IV, with no differences between groups in the frequency of shifts or the number of transitions to or from a specific CST group.

### 3.2. Safety Management

Adverse events are shown in the tables according to the MedDRA classification, version 26.0 (see Table 5).

During the clinical trial, a total of 24 adverse events (AEs) were reported by 16 patients (active intervention = 10 events; placebo = 14 events). Two of these were serious adverse events: a gastric ulcer (placebo group) and a prolongation of hospitalization that led to inclusion in the study (active intervention group). All patients recovered from the AEs without consequences. In terms of causality, 13 events (54.2%) were considered unrelated to the intervention, 5 (20.8%) were deemed unlikely related to the intervention, 5 (20.8%) were potentially related to the intervention, and 1 event (4.2%) was considered likely related to the intervention.

During the trial, the most frequent adverse events were as follows: gestational diabetes (N = 2; 8.3%), hospitalization due to threatened preterm birth (N = 3; 12.5%), and COVID-19 (N = 2; 8.3%).

No serious unexpected adverse event reaction (SUSAR) was reported during the trial.

## 4. Discussion

The primary aim of this study was to determine if vaginal probiotics containing multiple strains of *Lactobacillus* could effectively reduce preterm birth rates in pregnant women with a high risk of preterm birth. This research holds particular relevance, as preterm birth remains a leading cause of neonatal morbidity and mortality globally. In this trial, the comparison between the probiotic and placebo groups showed no significant differences in preterm birth rates. These findings support the results of earlier studies that also found no substantial benefit with probiotic use in this context [48,49,50,51], suggesting that, while probiotics benefit other health conditions, their efficacy in preventing preterm birth remains unclear. Likewise, no significant differences between groups were found in the analysis of perinatal outcomes, including gestational age at birth and neonatal health indicators, further supporting the initial conclusion [52].

Another critical aim of this study was to explore whether the use of probiotics could modify the composition of vaginal microbiota, given the well-established association between low levels of *Lactobacillus* and an increased risk of preterm birth. These results suggest that the vaginal microbiota in the late second and early third trimester may be relatively stable and resistant to external modulation, even with daily administration of a multi-strain probiotic. This microbial resilience may explain the lack of statistically and biologically significant differences between groups and underscores the complexity of intervening in established microbial ecosystems during pregnancy. These results are consistent with other studies that also failed to demonstrate a clear change in vaginal flora following a probiotic intervention [53]. This suggests that the probiotics used were not potent enough to shift the microbial balance, or the existing microbial environment was resilient to change.

A significant strength of this study is its focus on a specific high-risk population: pregnant women at a high risk of preterm birth. By targeting this group, this study aims to provide insights directly applicable to those most likely to benefit from effective interventions. This study has several limitations. First, the sample size for the microbiota sub-analysis was relatively small and limited to a single center. This was primarily due to organizational constraints and laboratory availability, as microbiota sampling and processing were only feasible at one site. Second, environmental, nutritional, and behavioral factors that could influence microbiota composition were not controlled for. Finally, the timing of the intervention, which was administered when microbial communities were likely already well established, may have reduced the potential for significant modulation.

Nonetheless, the safety profile of the probiotic was confirmed, with no significant adverse effects reported, suggesting that while probiotics may not prevent preterm birth, they are safe in pregnant women.

This underscores the complex and multifaceted nature of preterm birth, highlighting the existing gaps in understanding its underlying causes and effective prevention strategies. The findings of this study contribute to this body of knowledge by providing additional evidence that vaginal probiotics do not reduce preterm birth rates. However, future studies should be undertaken to investigate probiotics as a preventive treatment initiated during the first trimester of pregnancy. This further emphasizes the need for continued research to identify and clarify the mechanisms behind preterm birth and to develop effective interventions and preventive measures. Exploring other therapeutic approaches, possibly in combination with probiotics or entirely new interventions, might be necessary to make significant progress in the reduction in preterm birth rates. It is important to note that this intervention was designed specifically to address risks in the current pregnancy, not as a long-term or interpregnancy preventive approach.

## 5. Conclusions

Our study did not reveal any statistical differences in the rate of spontaneous preterm birth between pregnant women receiving probiotics and those receiving a placebo. Furthermore, following a regimen with a four-strain probiotic, no statistically significant differences in the vaginal microbiota of women were detected between both groups, although subtle trends were observed. These trends suggest a potential depletion of *Lactobacilli* populations, a factor often linked to an increased risk of spontaneous preterm birth. Importantly, no safety concerns were identified, indicating a good safety profile of the investigational product.

Although the probiotic intervention did not prove effective in reducing preterm birth in this high-risk symptomatic population, its safety and tolerability support further investigation in other clinical settings, such as earlier in pregnancy or in combination with established preventive strategies.

## Figures and Tables

**Table 1:** Patient demographics.

			Active Treatment (*n* = 101)	Placebo (*n* = 99)
Age (years) [mean (SD)]			32.08 (5.70)	31.66 (6.28)
BMI (kg/m^2^) [mean (SD)]			24.93 (6.51)	23.34 (4.06)
Gestational age at inclusion [mean (SD)]			28.43 (2.71)	28.77 (2.66)
Ethnicity	White		65 (65%)	63 (63.6%)
	Hispanic		21 (21%)	19 (19.8%)
	Black		3 (3%)	2 (2.1%)
	Asian		3 (3%)	2 (2.1%)
	Other		8 (8%)	3 (3%)
Current smoking	Yes		17 (17%)	5 (5.1%)
	No		83 (83%)	93 (94.9%)
Parity	Term	0	66 (65.3%)	61 (61.6%)
		1	25 (24.8%)	32 (32.3%)
		>2	10 (9.9%)	6 (6.1%)
	Preterm	0	77 (76.2%)	86 (86.9%)
		1	20 (19.8%)	11 (11.1%)
		>2	4 (4%)	2 (2%)
	Abortions	0	52 (51.5%)	53 (53.5%)
		1	20 (19.8%)	11 (11.1%)
		>2	29 (28.7%)	16 (16.2%)
	Live	0	50 (49.5%)	52 (52.5%)
		1	32 (31.7%)	38 (38.4%)
		>2	15 (14.9%)	9 (9.1%)

**Table 2:** Distribution of characteristics at hospital discharge after birth.

	Active Treatment (*n* = 91)	Placebo (*n* = 90)	OR (95% CI)	*p*-Value
Gestational age at delivery (weeks) [mean (SD)]	36.33 (3.57)	37.20 (2.73)		0.1379
Preterm birth < 37 weeks	28 (32.9%)	22 (25.9%)	**1.37 (0.71 to 2.65)**	0.3125
Preterm birth < 34 weeks	16 (17.8%)	8 (9%)	**2.19 (0.88 to 5.40)**	0.0844
Preterm birth < 32 weeks	11 (12.2%)	3 (3.3%)	**4.07 (1.10 to 15.15)**	0.0260
Preterm birth < 28 weeks	6 (6.7%)	1 (1.1%)	**6.28 (0.74 to 53.28)**	0.1177
Preterm birth < 24 weeks	2 (2.2%)	0 (0%)	-	0.4972

**Table 3:** Distribution of neonatal morbidity. (NICU, neonatal intensive care unit).

	Active Treatment (*n* = 91)	Placebo (*n* = 90)	OR (95% CI)	*p*-Value
Weight at birth (mean ± SD)	2799.87 (751.78)	2874.67 (661.72)	-	0.6699
Composite outcome	32 (35.3%)	29 (32.2%)	**1.14 (0.62 to 2.11)**	0.6754
Low birth weight (<2500 g)	8 (8.9%)	4 (4.4%)	**2.07 (0.60 to 7.14)**	0.2320
NICU admission	18 (22.5%)	15 (17.4%)	**1.23 (0.58 to 2.63)**	0.4145
Hyaline membrane	10 (55.6%)	3 (21.4%)	**3.58 (0.95 to 13.47)**	0.0512
Brain hemorrhage	1 (5.6%)	2 (14.3%)	**0.49 (0.04 to 5.49)**	0.5681
Neonatal sepsis	4 (22.2%)	1 (7.1%)	**4.09 (0.45 to 37.35)**	0.3547
Necrotizing enterocolitis	0 (0%)	2 (14.3%)	-	0.1835

**Table 4:** Community state type (CST) frequency in both groups after the intervention.

			Treatment Received		
Characteristic	*n*	Overall, *n* = 55 ^1^	Probiotics, *n* = 30	Placebo, *n* = 25	*p*-Value ^2^
**CST Before**, *n* (%)	51				0.69
CST-I		13 (25%)	8 (29%)	5 (22%)	
CST-II		5 (9.8%)	2 (7.1%)	3 (13%)	
CST-III		23 (45%)	14 (50%)	9 (39%)	
CST-IV		9 (18%)	4 (14%)	5 (22%)	
CST-V		1 (2 %)	0 (0%)	1 (4.3%)	
**CST After**, *n* (%)	41				0.80
CST-I		8 (20%)	5 (21%)	3 (18%)	
CST-II		4 (9.8%)	2 (8.3%)	2 (12%)	
CST-III		17 (41%)	11 (46%)	6 (35%)	
CST-IV		12 (29%)	6 (25%)	6 (35%)	
CST-V		0 (0%)	0 (0%)	0 (0%)	
**CST shift**, *n* (%)	37				>0.99
No		31 (84%)	18 (82%)	13 (87%)	
Yes		6 (16%)	4 (18%)	2 (13%)	
**Transition from specific CST**, *n* (%)	6				0.20
CST-I. Yes		2 (33%)	2 (50%)	0 (0%)	
CST-II. Yes		1 (17%)	0 (0%)	1 (50%)	
CST-III. Yes		2 (33%)	2 (50%)	0 (0%)	
CST-V. Yes		1 (17%)	0 (0%)	1 (50%)	
**Transition to specific CST**, *n* (%)	6				>0.99
CST-I. Yes		1 (17%)	1 (25%)	0 (0%)	
CST-IV. Yes		5 (83%)	3 (75%)	2 (100%)	

^1^ *n* (%). ^2^ Fisher’s exact test.

**Table 5:** Adverse effects.

Adverse Effect Number	Group	PT Name	PT Code	SOC Name	SOC Code	Intensity	Causality	SAE?
1	Placebo	Gastric ulcer	10017822	Gastrointestinal disorders	10017947	Moderate	Not related	Yes
2	Active treatment	Hospitalization	10054112	Surgical and medical procedures	10042613	Mild	Not related	Yes
3	Active treatment	Amniotic cavity infection	10060937	Infections and infestations	10021881	Mild	Not related	No
4	Placebo	Gestational diabetes	10018209	Pregnancy, puerperium, and perinatal conditions	10036585	Mild	Not related	No
5	Placebo	Abdominal Pain	10000081	Gastrointestinal disorders	10017947	Mild	Possible	No
6	Placebo	Constipation	10010774	Gastrointestinal disorders	10017947	Mild	Possible	No
7	Placebo	Uterine contractions during pregnancy	10049975	Pregnancy, puerperium, and perinatal conditions	10036585	Mild	Possible	No
8	Placebo	Genital discharge	10056740	Reproductive system and breast disorders	10038604	Mild	Possible	No
9	Placebo	Photopsia	10034962	Eye disorders	10015919	Mild	Unlikely	No
10	Placebo	Edema peripheral	10030124	General disorders and administration site conditions	10018065	Mild	Unlikely	No
11	Placebo	Suprapubic pain	10050822	General disorders and administration site conditions	10018065	Mild	Possible	No
12	Active treatment	Dyspepsia	10013946	Gastrointestinal disorders	10017947	Mild	Not related	No
13	Active treatment	Abdominal Pain upper	10000087	Gastrointestinal disorders	10017947	Moderate	Probable	No
14	Active treatment	Fetal growth restriction	10070531	Pregnancy, puerperium, and perinatal conditions	10036585	Mild	Not related	No
15	Placebo	Premature delivery	10036595	Pregnancy, puerperium, and perinatal conditions	10036585	Moderate	Unlikely	No
16	Placebo	Threatened labor	10043508	Pregnancy, puerperium, and perinatal conditions	10036585	Mild	Unlikely	No
17	Active treatment	Preeclampsia	10036485	Pregnancy, puerperium, and perinatal conditions	10036585	Mild	Not related	No
18	Active treatment	Urinary tract infection	10046571	Infections and infestations	10021881	Mild	Not related	No
19	Active treatment	Gestational diabetes	10018209	Pregnancy, puerperium, and perinatal conditions	10036585	Mild	Not related	No
20	Active treatment	COVID-19	10084268	Infections and infestations	10021881	Mild	Not related	No
21	Active treatment	Threatened labor	10043508	Pregnancy, puerperium, and perinatal conditions	10036585	Mild	Not related	No
22	Placebo	Threatened labor	10043508	Pregnancy, puerperium, and perinatal conditions	10036585	Mild	Not related	No
23	Placebo	Adverse drug reaction	10061623	General disorders and administration site conditions	10018065	Mild	Not related	No
24	Placebo	COVID-19	10084268	Infections and infestations	10021881	Mild	Unlikely	No

PT (preferred term). SOC (system organ class), SAE (Serious Adverse Event).

## Data Availability

The original contributions presented in this study are included in the article. Further inquiries can be directed to the corresponding author.

## Abbreviations

PTB	preterm birth
CSTs	composted tannery sludge
sPTB	spontaneous preterm birth
AEs	adverse events
SUSAR	serious unexpected adverse event reaction
BMI	body mass index
